# Microbial dysbiosis and inferred functional profiling reveals the potential role of *Methylobacterium* in prostate cancer

**DOI:** 10.3389/fcimb.2026.1760700

**Published:** 2026-06-17

**Authors:** Zainab M. Al Shareef, Rula M. Al-Shahrabi, Fatemeh Saheb Sharif-Askari, Burcu Yener, Poorna M. Bhamidimarri, Amal Bouzid, Iman M. Talaat, Riyad Bendardaf, Rifat A. Hamoudi, Sambhaji Mote, Raghvendra Mall, Filippo Castiglione

**Affiliations:** 1 Department of Basic Medical Sciences, College of Medicine, University of Sharjah, Sharjah, United Arab Emirates; 2 Sharjah Institute for Medical Research, University of Sharjah, Sharjah, United Arab Emirates; 3 Department of Pharmacy Practice and Pharmacotherapeutics, College of Pharmacy, University of Sharjah, Sharjah, United Arab Emirates; 4 Clinical Sciences Department, College of Medicine, University of Sharjah, Sharjah, United Arab Emirates; 5 Pathology Department, Faculty of Medicine, Alexandria University, Alexandria, Egypt; 6 Oncology Unit, University Hospital of Sharjah, Sharjah, United Arab Emirates; 7 Division of Surgery and Interventional Science, University College London, London, United Kingdom; 8 Biotechnology Research Centre, Technology Innovation Institute, Abu Dhabi, United Arab Emirates; 9 Qatar Computing Research Institute, Hamad Bin Khalifa University, Doha, Qatar; 10 Institute for Applied Computing, National Research Council of Italy, Rome, Italy

**Keywords:** Gleason, metagenomics, methylobacterium, prostate cancer, UAE

## Abstract

**Background and objective:**

Prostate cancer (PCa) is a leading malignancy in men, with a multifactorial aetiology involving genetic, hormonal, and microbial factors. Although emerging evidence implicates tumour-associated microbial communities in cancer biology, microbial signatures in PCa, particularly in Arab populations, remain underexplored. This study aimed to characterize the prostate tissue microbiota in an Arab cohort and explore associations with clinical features.

**Methods:**

In this retrospective study, 40 formalin-fixed paraffin-embedded (FFPE) prostate tissue samples (23 PCa and 17 benign prostatic hyperplasia [BPH]) were analysed using 16S rRNA gene sequencing. Microbial diversity, taxonomic composition, and predicted functional potential inferred from 16S data were assessed using DADA2 (v1.30.0), phyllode (v1.46.0), and PICRUSt2 (v2.5.2), with taxonomic classification based on the SILVA database (release 138). Beta diversity differences were tested using PERMANOVA (999 permutations), and differential abundance analyses were corrected using false discovery rate (FDR).

**Key findings and limitations:**

PCa tissues demonstrated higher alpha diversity than BPH samples, with greater heterogeneity in beta diversity. Among the identified genera, *Methylobacterium* was enriched in PCa samples and remained directionally consistent after multivariable adjustment. Exploratory analyses suggested higher abundance in advanced and deceased cases; however, survival findings were limited by sample size. Functional inference indicated enrichment of predicted pathways for carbohydrate and nitrogen metabolism.

**Conclusions:**

This exploratory study identified *Methylobacterium* as a candidate microbial signature associated with PCa in an Arab cohort. Given the modest sample size and the inferential nature of functional predictions, these findings require validation in larger prospective studies using direct metagenomic and metabolomic approaches.

## Introduction

Prostate cancer (PCa) is one of the most commonly diagnosed cancers in men worldwide (Siegel et al., 2024). The pathogenesis involves a complex interplay of genetic, hormonal, environmental, and potentially microbial factors. Advances in human microbiome research have increasingly implicated microbial communities in cancer biology, including PCa (Berenguer et al., 2023; Kim et al., 2024).

Metagenomic approaches, particularly 16S rRNA gene sequencing, have enabled the characterization of microbial ecosystems and elucidation of their functional roles in various cancers (Turnbaugh et al., 2007; Thomas et al., 2012). These approaches have demonstrated reproducibility across populations and potential utility in identifying disease-associated microbial patterns. For example, a multinational study conducted in Japan, Spain and Germany reported that microbial signatures achieved high predictive accuracy among different ethnic groups (Nagata et al., 2022).

In a healthy genitourinary microbiome, balanced microbial diversity supports immune regulation and limits overgrowth of pathogenic bacteria. Dysbiosis within this ecosystem has been associated with chronic inflammation, immune modulation, and processes implicated in PCa initiation and progression (Sfanos et al., 2018b; Nicolaro et al., 2020). Several studies have reported differences in microbial composition between PCa and benign prostatic hyperplasia (BPH), suggesting potential relevance for disease stratification (Ward Grados et al., 2024). However, most existing investigations have focused on Western and East Asian populations, limiting generalizability to Middle Eastern and Arab cohorts (Feng et al., 2019b; Kim et al., 2024).

Human microbiome composition and diversity vary significantly across ethnic and geographic populations (Gupta et al., 2017; Feng et al., 2019a). Our previous work in the United Arab Emirates (UAE) identified notable microbial diversity differences between local and South Asian BPH patients (Al Shareef et al., 2022)., underscoring the importance of population-specific investigations. Despite this, Arab cohorts remain largely underrepresented in PCa microbiome research.

In the present study, we applied 16S rRNA gene sequencing with predictive functional analysis to analyse formalin-fixed paraffin-embedded (FFPE) prostate tissue samples from PCa and BPH patients of Arab ethnicity in the UAE. We characterised microbial composition and diversity and explored associations with prostate-specific antigen (PSA) levels, Gleason scores, and survival outcomes. Given the retrospective design and modest sample size, these analyses are intended to provide exploratory insights and generate hypotheses for future validation in larger, prospective cohorts.

## Materials and methods

### Clinical specimens

In this study, 23 PCa and 17 BPH FFPE prostate tissues were obtained from the archive of prostate cases at the UHS pathology laboratory from 2011 to 2022. The tissues were selected based on the physician’s diagnosis and histopathology confirmation. Patient demographic data, along with pathological markers (prostate size, PSA, Gleason score), were collected. Detailed patient information is provided in **Supplementary Table 1**.

Given the low microbial biomass nature of FFPE tissue samples, strict laboratory precautions were implemented to minimize environmental and reagent contamination. DNA extraction blank controls and PCR negative controls were processed in parallel with all clinical samples throughout DNA extraction and amplification procedures to monitor background contamination.

### DNA extraction and sequencing of bacterial 16S rRNA

DNA was extracted from five to six consecutive 5 μm sections of FFPE tissue biopsies from PCa and BPH samples using QIAamp DNA tissue extraction kit (Qiagen, Hilden, Germany). According to the manufacturer’s instructions.

The quantity and purity of the extracted DNA were measured using NanoDrop2000 (Thermo Fisher Scientific, Waltham, MA, USA). Primers were selected from the literature to cover conserved regions spanning the variable regions V1-V8 of the bacterial 16S rRNA gene, generating amplicons ranging from 103 to 217 base pairs (bp). Primer details are provided in **Supplementary Table 2** and have been previously described elsewhere (Ludwig et al., 1993; Muyzer et al., 1993; Chakravorty et al., 2007; D’Amore et al., 2016; Inagaki et al., 2016; Winand et al., 2019). Library preparation was initiated with 10 ng of input DNA, ensuring sufficient template for amplification. A primer mix was prepared using target-specific primers (100 µM) at a final concentration of 10 µM each.

Polymerase chain reaction (PCR) amplification was performed using the FastStart^®^ High Fidelity PCR System (Roche, Basel, Switzerland) under previously described thermal cycling conditions (Gouda et al., 2022). Amplified products were purified using ExoSAP-IT (Invitrogen, Carlsbad, CA, USA) and diluted with nuclease-free water. The purified amplicons were re-amplified with barcode incorporation using FastStart High Fidelity Master Mix. Libraries were purified using AMPure XP beads (Beckman Coulter, Brea, CA, USA) and quantified using 6% acrylamide gel electrophoresis and the Agilent High Sensitivity DNA Bioanalyzer assay (Agilent Technologies, Santa Clara, CA, USA).

The libraries were diluted to 100 pM and sequenced on the Ion S5 XL semiconductor sequencer using the Ion 520 Chip (Life Technologies Corporation, Carlsbad, CA) prepared on the fully automated Ion Chef System (Thermo Fisher Scientific, Waltham, MA, USA) according to the manufacturer’s instructions.

Extraction blank controls and PCR negative controls yielded negligible amplification (441 post-QC reads across 11 sequences; **Supplementary Table 3A**). Taxa detected in the NTC were cross-referenced against the patient OTU matrix. Taxa absent from all patient samples (Aquirufa, Dictyoglomus) were excluded. Enterobacter, detected in the NTC at 36 reads, was excluded based on its substantially higher abundance in the E. coli positive control (6,692 reads), confirming it represents reagent background; a conservative threshold of ≥40 NTC reads was applied for exclusion.

### Analysis

Raw FASTQ files were processed using DADA2 (v1.30.0) implemented in R (v4.5.1) to denoise the single-end reads and generate amplicon sequence variants (ASVs). Quality filtering was applied before denoising, and chimeric sequences were removed using the consensus method within DADA2.

Taxonomic classification was performed using the SILVA reference database (release 138), replacing the previously used GreenGenes database to improve taxonomic resolution and reproducibility.

To address potential contamination in this low-biomass dataset, all taxa detected in the NTC extraction blank were manually cross-referenced against the patient OTU matrix. Taxa present in the NTC but absent from patient samples (Aquirufa, Dictyoglomus) were excluded. Enterobacter was excluded using a conservative NTC count threshold of ≥40 reads, justified by its substantially higher abundance in the E. coli positive control (6,692 reads versus 36 reads in the NTC), confirming the NTC signal represents reagent contamination rather than biological carryover. *Methylobacterium and Bradyrhizobium*, the two flagship taxa, were completely absent from the NTC (0 reads), supporting their classification as genuine tissue-associated biological signals (**Supplementary Tables 3A, S3B**).

The phyloseq package (v.1.46.0) was used to construct the abundance matrix and perform diversity analysis. Alpha diversity was assessed using the Shannon index. Beta diversity was calculated using Bray-Curtis dissimilarity and visualised by non-metric multi-dimensional scaling (NMDS). Group differences in beta diversity were tested using permutational multivariate analysis of variance (PERMANOVA) with 999 permutations (Douglas et al., 2020).

Differential abundance testing was conducted at the genus level, and p-values were adjusted for multiple comparisons using the Benjamini–Hochberg false discovery rate (FDR) correction.

For functional prediction, PICRUSt2 (v2.5.2) was used to infer KEGG orthologs, enzyme classes, and metabolic pathways from 16S rRNA gene data. These analyses estimate predicted functional potential based on phylogenetic inference and do not represent direct measurement of metagenomic capacity; therefore, functional findings are considered hypothesis-generating and require validation using shotgun metagenomics or metabolomic approaches.

## Results

A total of 40 FFPE samples were analysed, comprising 23 PCa and 17 BPH cases. Clinical characteristics, including age, PSA levels, prostate size, and Gleason scores, are summarized in **Supplementary Table 1**. The PCa group had significantly higher mean age and PSA levels than the BPH group (P < 0.05). Most PCa cases were diagnosed with advanced Gleason scores (≥8, P < 0.001). Representative histopathological differences between benign and malignant prostate tissues are illustrated in **Supplementary Figure 1**.

**Figure 1:**
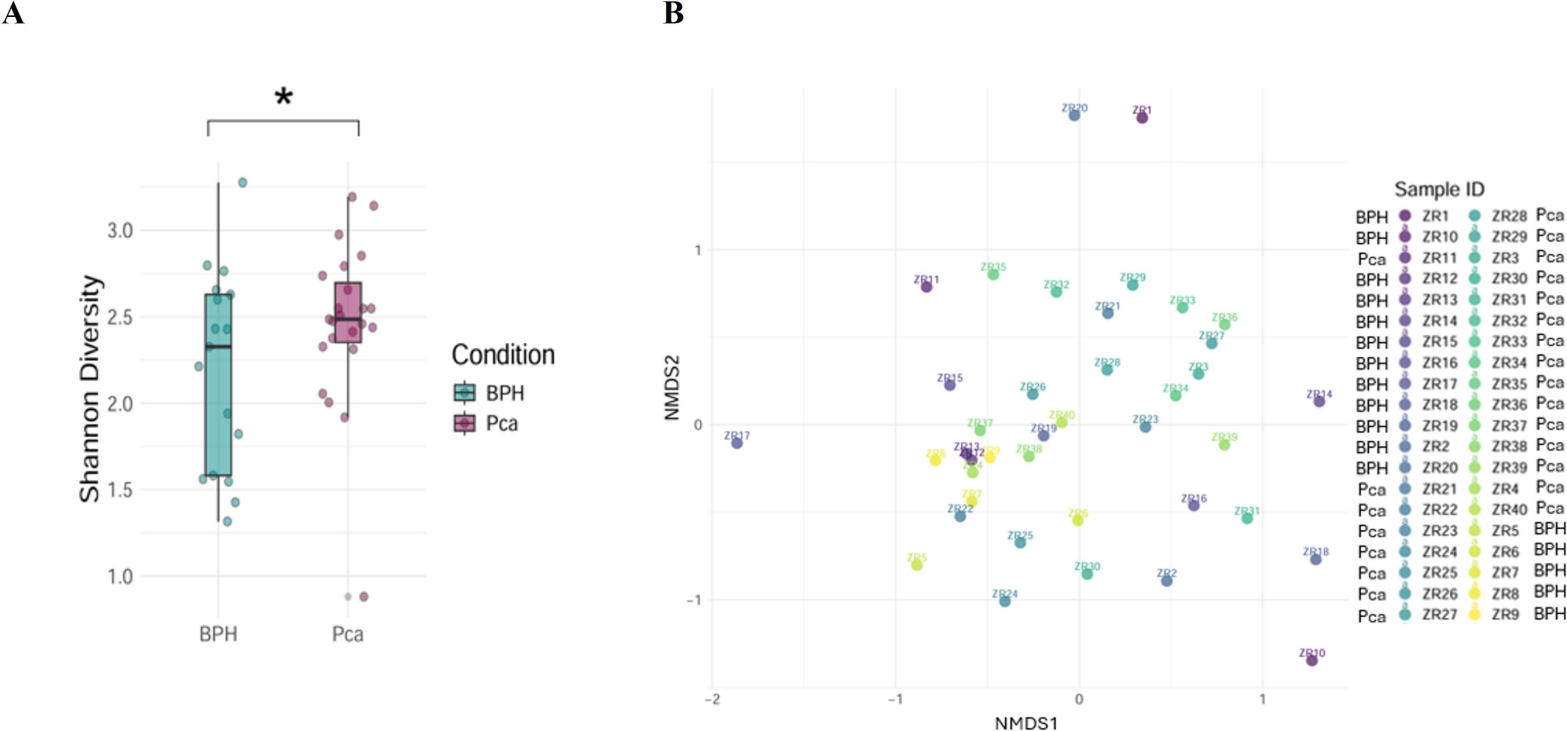
**(A)** Boxplot of Alpha diversity measured using the Shannon index, **(B)** non-metric multidimensional scaling (NMDS) analysis illustrating the Beta diversity of bacterial communities in BPH and PCa samples. The asterisk (*) denotes statistical significance at p < 0.05

Microbial diversity analysis revealed significantly higher alpha diversity (Shannon index) in PCa samples compared with BPH (P < 0.05). In contrast, BPH samples exhibited greater beta-diversity heterogeneity. Differences in microbial composition between groups were confirmed using PERMANOVA based on Bray-Curtis dissimilarity (999 permutations) (**Figures 1A, B**).

**Figure 2:**
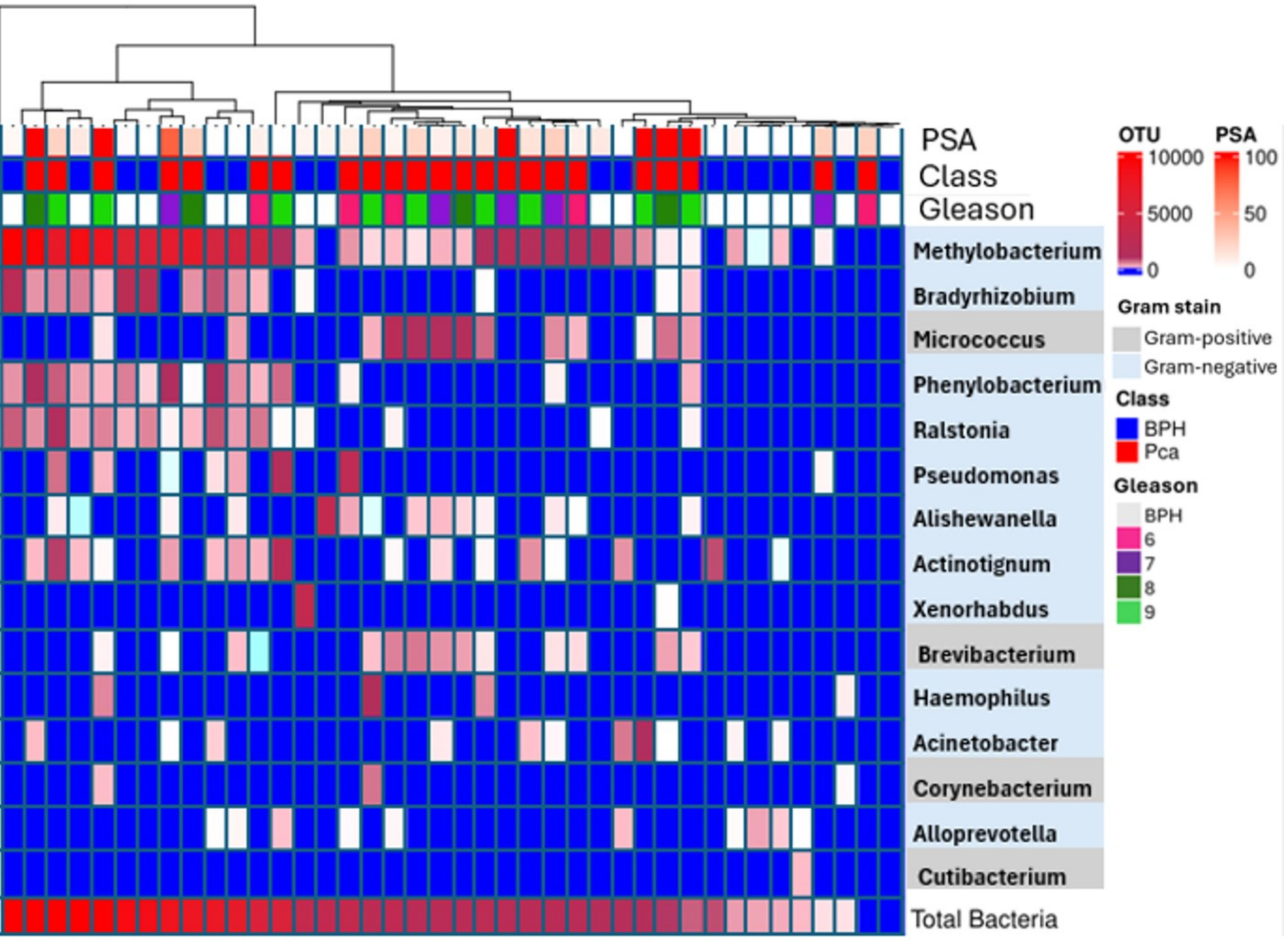
Clustering heatmap illustrating the abundance of selected bacterial genera in prostate tissue samples. Samples are annotated by PSA levels, clinical classification (BPH in blue, Pca in red), and Gleason scores (ranging from 6 to 9, colour-coded). OTU abundance levels range from low (blue) to high (red). Bacteria are distinguished based on Gram staining (Gram-positive: grey shaded, Gram-negative: blue shading). Total Bacteria row summarizes overall bacterial abundance across samples.

Taxonomic profiling identified 256 bacterial genera across all samples. Among these, *Methylobacterium, Bradyrhizobium*, and *Micrococcus* were enriched in PCa tissues (**Figure 2**). Gram-negative bacteria predominated in both groups. Genus-level comparisons between PCa and BPH samples are presented in **Supplementary Figure 2**. Differential abundance analyses were adjusted for multiple testing using the Benjamini–Hochberg false discovery rate (FDR).

**Figure 3:**
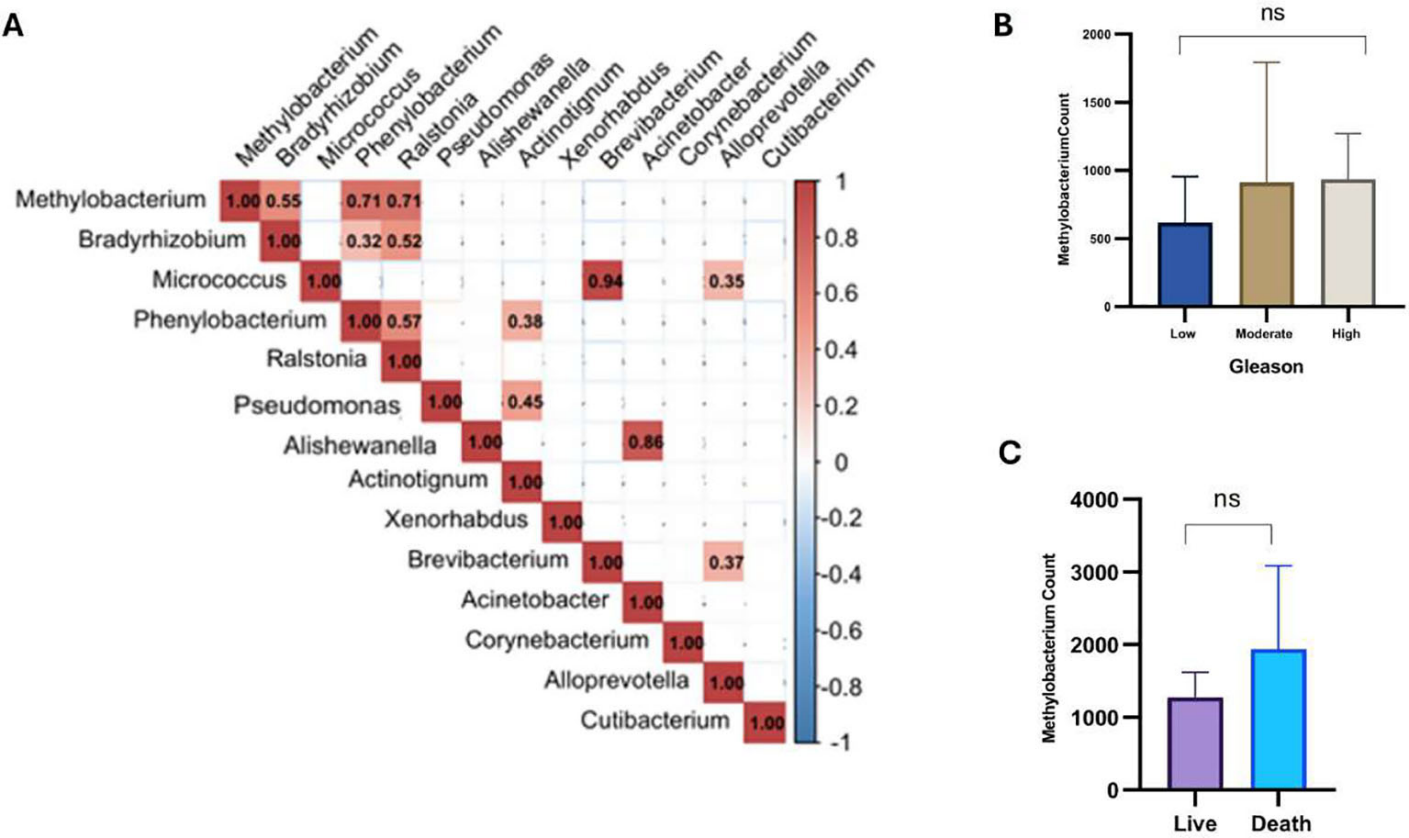
**(A)** Heatmap showing correlation coefficients between different bacterial genera identified in PCa samples. Values range from -1 (negative correlation, blue) to +1 (positive correlation, red), with darker colours indicating stronger correlations. **(B)** Comparison of the abundance of *Methylobacterium* across PCa samples grouped by Gleason score categories (Low, Moderate, High). No significant difference (ns) was observed among groups. **(C)** Comparison of *Methylobacterium* abundance between patients who survived (live) and those who died (deceased). No statistically significant difference was observed between groups (ns; unpaired t-test).

**Figure 4:**
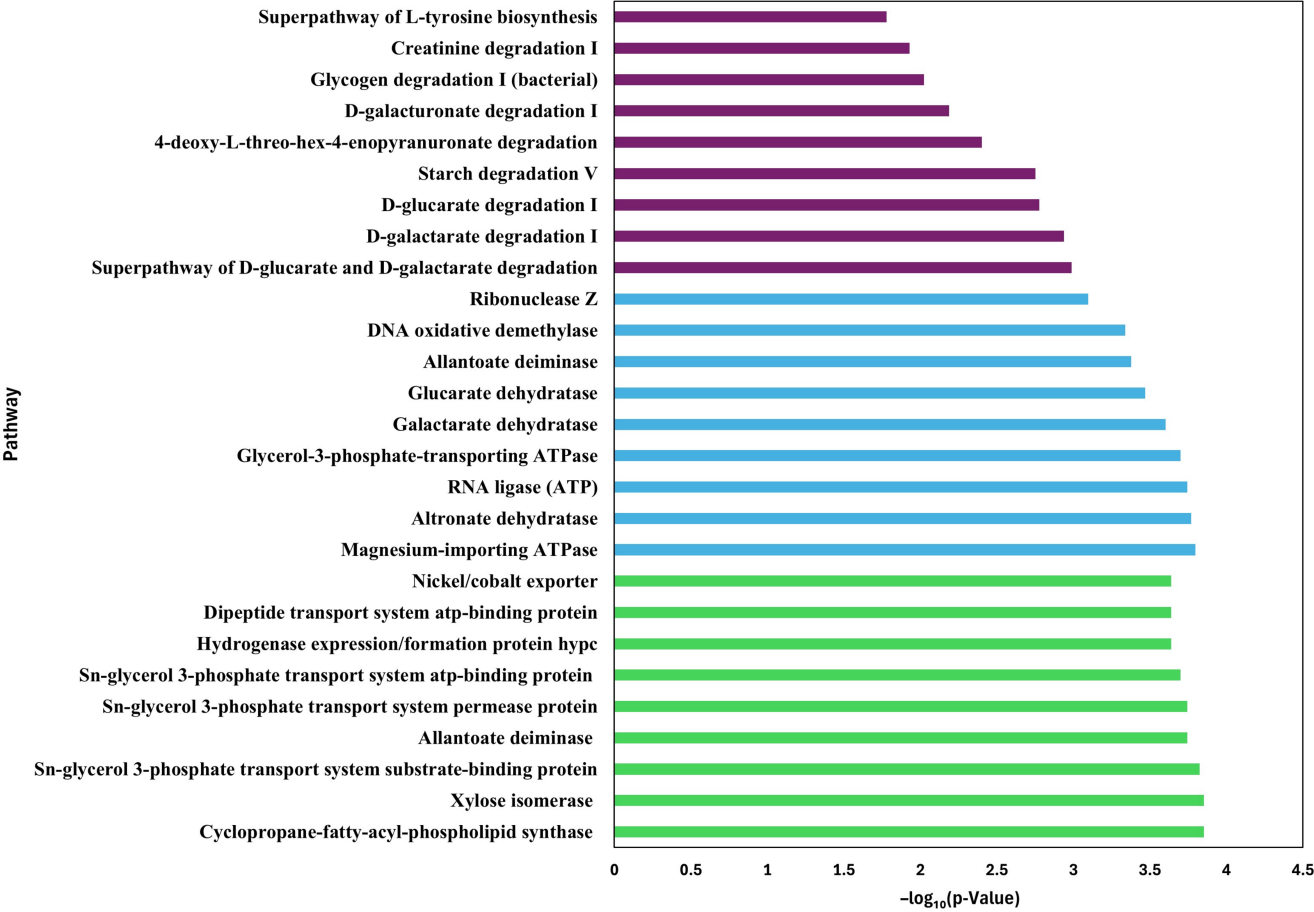
The –log_10_ (p-value) of enriched microbial functions identified in PCa tissue samples. Functions are classified into three major categories: Metabolic Pathways (purple), Enzyme Classifications (blue), and KEGG Orthologs (green).

Correlation matrix analysis demonstrated co-occurrence patterns among several genera, particularly *Methylobacterium, Bradyrhizobium, and Phenylobacterium* (**Figure 3A**). Although *Methylobacterium* abundance did not differ significantly across Gleason categories, higher abundance was observed in high-grade and deceased PCa patients; however, these differences were not statistically significant (p= NS; one-way ANOVA for Gleason groups; unpaired t-test for survival status; **Figures 3B, C**). Predicted functional pathway analysis (PICRUSt2 inference) indicated enrichment of carbohydrate and nitrogen metabolism pathways, including galactarate and creatinine metabolism, as well as increased microbial membrane transport activity in PCa samples (**Figure 4**). Enzyme class predictors showed higher representation of ATPases, dehydratases, and ligases. KEGG annotations suggested enrichment of stress-related and transport-associated functions in PCa compared to BPH. These functional findings are based on inference from 16S rRNA data and should be considered hypothesis-generating.

## Discussion

We conducted an exploratory microbiota analysis to investigate microbial composition, diversity, and predicted functional potential in PCa and BPH tissues within the Arab cohort. To our knowledge, few studies have characterised prostate tissue microbiota in this population. Our findings suggest distinct microbial patterns associated with PCa; however, given the retrospective design and modest sample size, these observations should be interpreted cautiously and not as evidence of causality or prognostic value.

Alpha diversity was significantly higher in PCa samples compared with BPH, while BPH exhibited greater beta diversity heterogeneity. Increased alpha diversity in tumour tissue has been variably reported in prior studies, with some cohorts demonstrating similar findings (Smith et al., 2021)., while others have reported no significant differences (Cavarretta et al., 2017; Banerjee et al., 2019; Feng et al., 2019b; Huang et al., 2024). Such discrepancies likely reflect differences in population background, sampling approach, tissue handling, and analytical methods. In our cohort, the observed increase in alpha diversity may reflect greater species richness and a more uniform microbial distribution within tumour-associated tissue. However, mechanistic implications cannot be inferred from these data.

Several genera, including Methylobacterium, Micrococcus, Bradyrhizobium, Pseudomonas, and Acinetobacter, demonstrated differential abundance patterns between PCa and BPH. Many of these genera are commonly identified within genitourinary microbiota and have been reported in both commensal and opportunistic contexts (Aragón et al., 2018). The tumour microenvironment in PCa is characterized by chronic inflammation, immune modulation, and altered metabolic states (Sfanos et al., 2018b; Sfanos et al., 2018a) which may influence microbial colonization patterns. Additionally, exposure to hormonal or therapeutic interventions may reshape microbial composition (Sfanos et al., 2018a; Zhong et al., 2022). However, whether these microbial shifts represent drivers, passengers, or consequences of tumour-associated changes remains unclear and requires further investigation.

*Methylobacterium* enrichment in PCa tissues was one of the most consistent observations in this study. Prior work has reported the presence of *Methylobacterium* species in PCa biopsies in an Indian cohort (Sarkar et al., 2022). However, this genus has been described as an opportunistic pathogen in immunocompromised individuals (Kovaleva et al., 2014; Cao et al., 2024). Its biological role in PCa remains poorly defined. In our cohort, *Methylobacterium* abundance appeared numerically higher in high-grade and deceased cases;. However, these differences were not statistically significant (P = NS) and should be interpreted cautiously given the small number of events. Therefore, no prognostic conclusions can be drawn.

Reports in other malignancies, including gastric and breast cancer, suggest that *Methylobacterium* may be associated with altered immune or microenvironmental states (Xuan et al., 2014; Yazdi et al., 2016; Wang et al., 2025). Nonetheless, such associations are context-specific and cannot be extrapolated directly to PCa. At present, *Methylobacterium* should be considered a candidate microbial signature requiring validation rather than a biomarker or mechanistic driver of tumour progression.

Although Kim et al. (2023) reported a high prevalence of *Methylobacterium* in PCa patients, its enrichment was most notable in the non-recurrent PCa group compared with recurrent PCa, indicating microbial richness without a clear association with disease progression (Kim et al., 2023). However, *Methylobacterium* species have been reported to be more prevalent in breast cancer, suggesting a potential association with cancerous environments (Xuan et al., 2014; Yazdi et al., 2016). While *Methylobacterium’s* role in PCa remains poorly understood, further research is needed to clarify its clinical significance and potential mechanistic involvement.

Predicted functional pathway analysis indicated enrichment of carbohydrate and nitrogen metabolism pathways in PCa tissues, as well as creatinine-related pathways. Sarcosine has previously been linked to PCa aggressiveness (Sreekumar et al., 2009). However, our functional findings are based on inference from 16S rRNA gene data (PICRUSt2) and do not represent direct measurement of microbial metabolic activity. Therefore, interpretations regarding metabolic modulation remain hypothetical and require validation using direct metabolomic or shotgun metagenomic approaches.

Beyond local tissue associations, increasing evidence suggests that tumour-associated microbiota may interact with systemic metabolic and inflammatory pathways. Microbiota–metabolome interactions have been associated with cardiometabolic and inflammatory phenotypes in other disease contexts (Jin et al., 2024; He et al., 2025; Tan et al., 2025; Zhou et al., 2025). While our study was not designed to assess systemic effects, the observed predicted pathway enrichment may reflect broader tumour–microenvironment interactions. Further integrative multi-omics studies are necessary to clarify host–microbe interactions in PCa.

Overall, our findings support the hypothesis that microbial composition differs between malignant and benign prostate tissues in this cohort. However, due to sample size constraints, retrospective sampling, and reliance on inference-based functional prediction, these findings should be regarded as exploratory and hypothesis-generating.

## Conclusion

This study characterizes the prostate tissue microbiota in an Arab cohort and identifies differential microbial patterns between PCa and BPH tissues. *Methylobacterium* was enriched in PCa samples and remained directionally consistent after adjustment for clinical variables; however, associations with tumour grade and mortality are exploratory and require validation.

Predicted enrichment of metabolic and transport-related pathways suggests potential tumour–microbiome interactions, but these findings are inference-based and do not establish causality.

Collectively, our results provide population-specific exploratory insights into the prostate tissue microbiota and highlight the need for larger prospective studies that integrate direct metagenomic and metabolomic profiling to clarify the biological relevance of these microbial signatures in prostate carcinogenesis.

## Data Availability

The datasets presented in this study can be found in online repositories. The names of the repository/repositories and accession number(s) can be found in the article/**Supplementary Material**. All raw metagenomic reads used in this study are available from NCBI under BioProject PRJNA1331457.
