# Supplementary material for: Microbial dysbiosis and inferred functional profiling reveals the potential role of Methylobacterium in prostate cancer
Source: Front Cell Infect Microbiol. 2026 Jun 17;16:1760700. doi: 10.3389/fcimb.2026.1760700 (PMC13318943; doi:10.3389/fcimb.2026.1760700)

Supplementary Material

# Supplementary Table 1: Clinical and pathological characteristics of patients with prostate cancer (PCa) and benign prostatic hyperplasia (BPH)

| **Variables** | **PCa** | | | **BPH** | **P -Value** |
| --- | --- | --- | --- | --- | --- |
| **Mean Age ± SEM** | **76 ±1.7** | | | **71±1.3** | **0.04** |
| **Mean PSA± SEM** | **53.29±8.2** | | | **4.83±0.65** | **0.02** |
| **Prostate size± SEM** | **57.14 ±6.9** | | | **64 ±10.5** | **0.6** |
| **Prostate density± SEM** | **0.83 ±0.2** | | | **0.11±0.01** | **0.344** |
| **Gleason score** | **Early≤7** | **Moderate 7 (4+3)** | **Advanced ≥8** | **NA** | **<0.001** |
|  | **7** | **3** | **13** |  |  |

Supplementary Table 1: The table summarizes the clinical and pathological features of patients diagnosed with PCa and BPH. Data are presented as mean ± standard error of the mean (SEM). Variables compared include age, prostate-specific antigen (PSA) levels, prostate size, and prostate density. Statistical significance for continuous variables was determined using the unpaired t-test; p-values <0.05 were considered statistically significant. Gleason scores are reported for PCa patients and categorized into early (≤7), moderate (7 [4+3]), and advanced (≥8) stages. Differences in Gleason score categories were assessed using one-way ANOVA, with p-values <0.05 considered significant.

**Supplementary Table 2: Primer Sequences, Product Sizes, and References for 16S rRNA Amplicon-Based Microbiota Profiling**

| **Name** | **Sequence** | **Product size** | **Reference** |
| --- | --- | --- | --- |
| V1_8F | AGAGTTTGATYMTGGCTCAG | 212 | (18) |
| V2_219R | AAGAGGCCCGAAGGTCCC |  | custom design |
| V3_334F | CCAGACTCCTACGGGAGGCAG | 204 | (19) |
| V3_537R | CGTATTACCGCGGCTGCTG |  | (19) |
| V4_806F | AACAGGATTAGATACCCTGGTAGTCC | 103 | (20) |
| V4_908R | CCCGTCAATTCMTTTGAGTT |  | (21) |
| V4_806F | AACAGGATTAGATACCCTGGTAGTCC | 121 | (20) |
| V5_926R | CCGTCAATTCCTTTRAGTTT |  | (20) |
| V4_908F | AACTCAAAKGAATTGACGGG | 168 | (20) |
| V5_1075R | CACGAGCTGACGACARCC |  | (20) |
| V6_961F | TCGATGCAACGCGAAGAA | 126 | (19) |
| V6_1085R | ACATTTCACAACACGAGCTGACGA |  | (19) |
| V6_931F | CGCACAAGCGGTGGAGCA | 131 | (22) |
| V6_1061R | CCTGTCTCACGGTTCCCG |  | (22) |
| V8_1243F | GAGGAAGGTGGGGATGACGT | 217 | (23) |
| V8_1459R | AGGCCCGGGAACGTATTCA |  | (23) |

**Supplementary Table 3A:** **Negative and Positive Control Read Count Summary**

| **Control type** | **Raw reads** | **Post-QC reads** | **OTUs** |
| --- | --- | --- | --- |
| Extraction blank (NTC) | 1,103 | 441 | 11 |
| Positive control (E. coli genomic DNA) | 2,436,802 | 2,436,802 | 18 |

**Supplementary Table 3B:** **Taxa Detected in NTC Extraction Blank**

| **OTU** | **Reads** | **% NTC** | **Order** | **Family** | **Genus** |
| --- | --- | --- | --- | --- | --- |
| 18 | 153 | 34.7% | Cytophagales | Flectobacillaceae | **Aquirufa** |
| 4+27 | 73 | 16.6% | Moraxellales | Moraxellaceae | Acinetobacter |
| 20 | 65 | 14.7% | — | — | Unclassified |
| 6 | 46 | 10.4% | Dictyoglomales | Dictyoglomaceae | **Dictyoglomus** |
| 21 | 36 | 8.2% | Enterobacterales | Enterobacteriaceae | **Enterobacter** |
| 22 | 35 | 7.9% | Hyphomicrobiales | — | Unclassified |
| 23 | 13 | 2.9% | Hyphomicrobiales | Nitrobacteraceae | Unclassified |
| 24 | 10 | 2.3% | — | — | Unclassified |
| 25 | 5 | 1.1% | Eubacteriales | Acutalibacteraceae | Acutalibacter |
| 26 | 5 | 1.1% | Caulobacterales | Caulobacteraceae | Phenylobacterium |
| 27 | 3 | 0.7% | Moraxellales | Moraxellaceae | Acinetobacter |
| **TOTAL** | **441** | **100%** |  |  |  |

# Supplementary Figures

## Supplementary Figure 1: Histopathological representation of selected benign and malignant prostate tissue stained with Hematoxylin and Eosin (H&E).


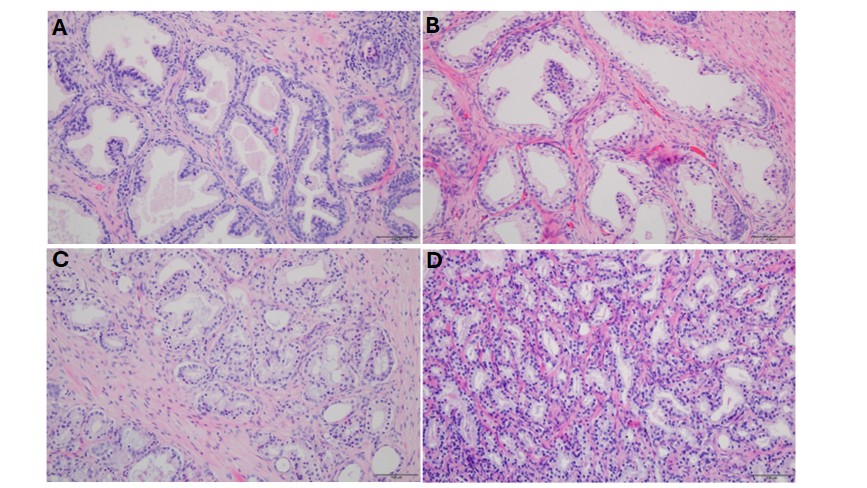


**Supplementary Figure 1** : illustrate the histopathological differences between benign and cancerous prostate tissue, stained with Hematoxylin and Eosin (H&E) and viewed under 20× magnification (scale bar = 100 μm). (A) and (B) show benign prostate tissue, characterized by well-organized and clearly defined glandular structures. (C) highlights a case of moderate PCa (Gleason score 3+4), where glandular structures appear irregular and partially fused. (D) represent advanced PCa (Gleason score 4+4), with severely disorganized and completely fused glands.

**2.2 Supplementary** **Figure 2:** **Differential genus-level microbial abundance between PCa and BPH tissues**


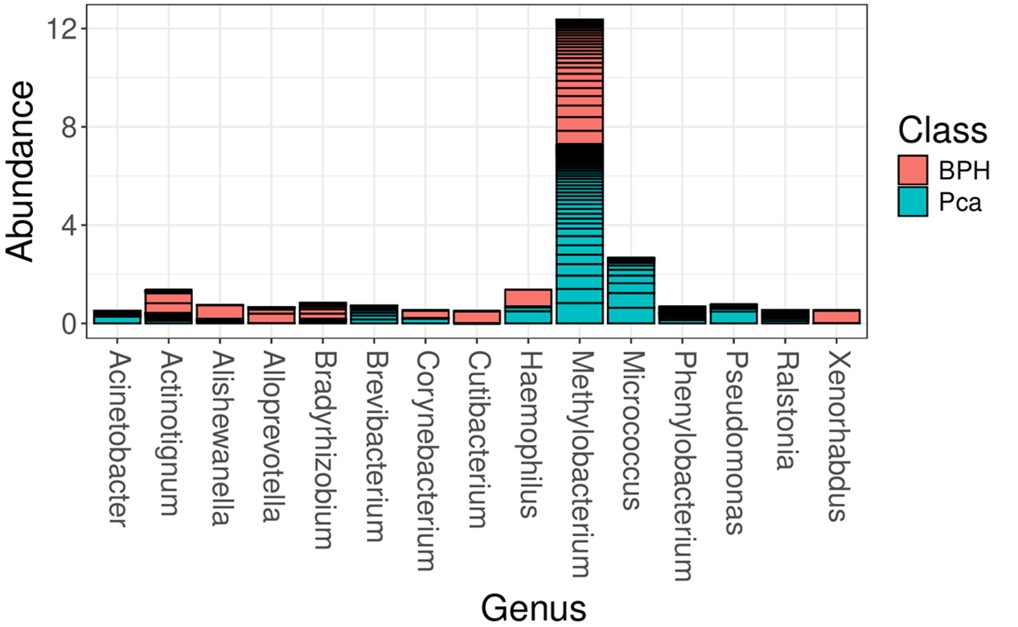

Supplement: Supplementary file 3 [file Table3.docx]
